# Comparison of Two Automated Targeted Metabolomics Programs to Manual Profiling by an Experienced Spectroscopist for ^1^H-NMR Spectra

**DOI:** 10.3390/metabo12030227

**Published:** 2022-03-04

**Authors:** Xiangyu Wang, Beata Mickiewicz, Graham C. Thompson, Ari R. Joffe, Jaime Blackwood, Hans J. Vogel, Karen A. Kopciuk

**Affiliations:** 1 Department of Mathematics and Statistics, University of Calgary, Calgary, AB T2N 1N4, Canada; s20090009@s.upc.edu.cn; 2 Department of Pediatrics, Cumming School of Medicine and Alberta Children’s Hospital Research Institute, University of Calgary, Calgary, AB T2N 4N1, Canada; bmmickie@ucalgary.ca; 3 Departments of Pediatrics and Emergency Medicine, Cumming School of Medicine, University of Calgary, Calgary, AB T2N 4N1, Canada; graham.thompson@albertahealthservices.ca; 4 Department of Pediatrics, Division of Pediatric Critical Care, University of Alberta, Edmonton, AB T6G 1C9, Canada; ari.joffe@albertahealthservices.ca; 5 Department of PICU and Critical Care, Alberta Children’s Hospital, Alberta Health Services, Calgary, AB T3B 6A8, Canada; jaime.blackwood@albertahealthservices.ca; 6 Department of Biological Sciences, Faculty of Science, University of Calgary, Calgary, AB T2N 1N4, Canada; 7 Departments of Community Health Sciences, Mathematics and Statistics, and Oncology, University of Calgary, Calgary, AB T2N 1N4, Canada; kakopciu@ucalgary.ca; 8 Cancer Epidemiology and Prevention Research, Alberta Health Sciences, Calgary, AB T2S 3C3, Canada

**Keywords:** BATMAN, ^1^H-NMR, manual profiling, Mnova, pediatric sepsis, rDolphin, targeted profiling

## Abstract

Automated programs that carry out targeted metabolite identification and quantification using proton nuclear magnetic resonance spectra can overcome time and cost barriers that limit metabolomics use. However, their performance needs to be comparable to that of an experienced spectroscopist. A previously analyzed pediatric sepsis data set of serum samples was used to compare results generated by the automated programs rDolphin and BATMAN with the results obtained by manual profiling for 58 identified metabolites. Metabolites were selected using Student’s *t*-tests and evaluated with several performance metrics. The manual profiling results had the highest performance metrics values, especially for sensitivity (76.9%), area under the receiver operating characteristic curve (0.90), precision (62.5%), and testing accuracy based on a neural net (88.6%). All three approaches had high specificity values (77.7–86.7%). Manual profiling by an expert spectroscopist outperformed two open-source automated programs, indicating that further development is needed to achieve acceptable performance levels.

## 1. Introduction

Metabolomics has increasingly been utilized in a number of domains, including medical settings, typically to characterize groups based on sets of metabolites that can be used to differentiate between them. Clinical applications generally require metabolites to be identified in order to better understand the disease process, and so targeted approaches are utilized [1,2]. One-dimensional (1D) proton nuclear magnetic resonance (^1^H-NMR) spectroscopy is a popular metabolomics platform for a number of reasons, such as high reproducibility, preservation of samples, short acquisition time, and quantification of metabolites over a dynamic range [3,4,5,6,7]. However, ^1^H-NMR-based metabolomics studies require substantial time and effort to obtain reliable identifications and quantifications of known metabolites through manual spectral fitting by an expert before any statistical analyses of the cohort can take place.

A number of automated, non-commercial programs have been developed over the past 10 years to reduce the time and resource requirements for the identification and quantification of NMR spectra as well as improve the reproducibility of the results. Some automated programs for targeted analyses include BQuant, AQuA, BATMAN, BAYESIL, ASICS, and Dolphin/rDolphin [5,6,8,9,10,11,12,13,14]. These programs employ different algorithms on a variety of platforms, including R, MATLAB, and web-based implementations. However, not all of these programs are still available, and if they are, they are being maintained and coded using open-source programs. Two programs that meet these requirements are BATMAN and rDolphin.

The Bayesian automated metabolite analyzer for ^1^H-NMR spectra (BATMAN) utilizes existing knowledge about the resonance signatures of peaks from publicly available databases, such as the Human Metabolome Database (HMDB) [15]. Known peaks are assigned to specific metabolites and quantified while unknown peaks are modelled using wavelets to incorporate potential features that contribute to understanding the studied phenomenon, for example, improving classification of the sample spectra. A Markov chain Monte Carlo algorithm estimates the joint posterior distribution of the parameters. The wavelet component is heavily penalized to favor known over unknown peaks in the likelihood during the burn-in phase of model fitting [16].

rDolphin implements the Dolphin program written in MATLAB (The Mathworks, Inc., Natick, Massachusetts) code in the open-source software program R [17]. Like Dolphin, rDolphin uses the Region of Interest (ROI) approach to identify peak areas where metabolites can be identified. The redesign of Dolphin into rDolphin addressed several limitations of Dolphin, providing improved visualization of spectral regions with high variability across sample spectra meriting scrutiny, options for enhanced metabolite identification, quality and reliability checks to minimize suboptimal quantification, and potential for novel metabolite identification. rDolphin includes a comprehensive graphical user interface (GUI) to facilitate exploratory analyses.

rDolphin and BATMAN share a number of program features which are implemented by different methodologies. Both programs use a targeted approach to identify metabolites and address peak overlap problems in 1D ^1^H-NMR spectra. In addition, both are written in the R programming language and utilize approaches to detect and potentially identify unknown metabolites or features. Lastly, both programs import information from the Human Metabolome Database (HMDB) for metabolite identification and quantification [15]. BATMAN is based on a Bayesian model that estimates model parameters from the posterior distribution, while rDolphin is based on a 1D line shape fitting approach.

The purpose of this pilot study was to compare results obtained by naïve operators who used these two automated, open-source programs (BATMAN and rDolphin) to carry out identification and quantification of 1D ^1^H-NMR metabolomics spectral data with the results obtained by manual profiling by an experienced spectroscopist. A targeted approach was needed for clinical diagnostic purposes in the original study [18], so this was also adopted here.

## 2. Results

The sensitivity was highest for the manual profiling method (76.9%), followed by the rDolphin and BATMAN methods (Table 1). Specificity for all three approaches was high and very similar (77.7–86.7%). Precision was highest for the manual profiling approach (62.5%) and lowest for the rDolphin method at 30.8%. The training accuracy was high for all three methods (>86.2%, results not shown). The testing accuracy showed a similar pattern to the sensitivity results, with the manual profiling performing very well (88.6%) and substantially lower results for BATMAN (58.3%) and rDolphin (48.9%). Terms are defined in the Methods section, below.

The average time needed to run all 173 samples in the rDolphin program on a laptop computer (Lenovo (Weifang, China) Intel(R) Core (TM) i5-7200U CPU @2.50 GHz, RAM:8 G) was 20 min. In contrast, BATMAN took over 40 min to complete 2500 runs using parallel computing on a server (Intel(R) Xeon(R) CPU E5 2620v3 @ 2.40 GHz 2.40 GHz RAM:128 G). The BioMark program took less than one minute to run each data set using the stability-based approach with the proportion selected set at 0.7. Each run for the MLPHM neural network took about 20 min, so 200 min in total.

**Figure 1:**
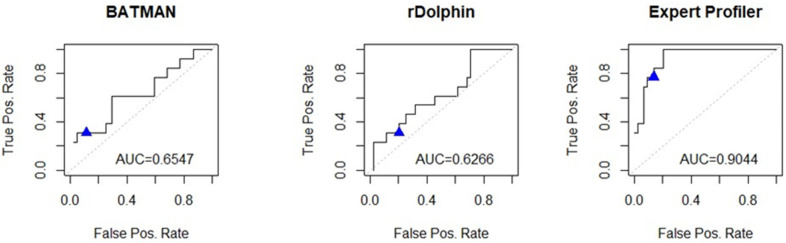
Area under the receiver-operating characteristic curve (AUC) calculated for each method: Expert Profiler, rDolphin, and BATMAN. The blue triangle represents the point estimate for the true-positive rate (sensitivity) and the false positive rate (1—specificity) based on the stability-based selection, using 0.7 from the BioMark package.

As shown in Figure 1, the area under the receiver operating characteristic curve (AUC) values based on the BioMark metabolite selections also varied substantially. The expert manual fitting had the highest AUC value of 0.90, followed by BATMAN with an AUC of 0.65 and rDolphin with an AUC of 0.63. This figure also reveals a similar point estimate for false-positive rates for all three approaches but substantially different sensitivities (true-positive rates).

**Figure 2:**
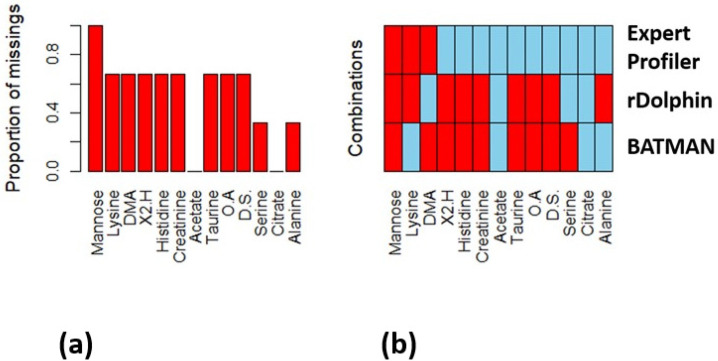
(**a**) Proportion of missings for all three methods combined showing which metabolites were more or less prone to not being correctly selected. (**b**) Heat map showing the patterns of selected (blue) and non-selected (red) correct metabolites for each method: Expert Profiler, rDolphin and BATMAN. Abbreviations: DMA = Dimethylamine, X2.H = 2-Hydroxyisovalerate, O.A = O-Acetylcholine, D.S. = Dimethyl sulfone.

Another way to represent the selection of top biomarkers is by a heat map. In Figure 2 the blue-colored cells demonstrate the selected metabolites for each method whereas the red-colored cells identify non-selected metabolites. The first six metabolites along the *x*-axis (left to right) are the ones with increased concentration in the pediatric intensive care unit (PICU) sepsis cohort rank, in order from the most significant (mannose) to the least significant (creatinine), while the last seven metabolites have decreased concentrations in this cohort, from most significant (acetate) to least significant (alanine).

## 3. Discussion

This study evaluated the use of two automated, open-source programs that carry out identification and quantification of 1D ^1^H-NMR metabolomics spectral data and compared their results with those obtained via manual profiling performed by an experienced spectroscopist using a commercially available program.

Our results revealed that the manual profiling results had the highest sensitivity compared with the rDolphin and BATMAN programs (Table 2). All three approaches had very similar and high specificity. Thus, for biomarkers that have been carefully validated, if specificity is more important than sensitivity in a clinical application, then the automated methods could work very well. For pediatric sepsis diagnosis and triage, a high specificity is not adequate, as being highly sensitive early on to detect the need for PICU is very important in making triage decisions. On this measure, the automated programs performed poorly, and this is reflected in the AUC results discussed next.

The AUC results showed the superiority of the manual profiling method, and the comparability of the BATMAN to the rDolphin program. Although the two automated programs had similar performance metrics, the selected metabolites for each were not the same. Both programs identified the same two metabolites from the decreased concentration in the PICU sepsis cohort, namely, acetate and citrate. rDolphin also identified serine whereas BATMAN identified alanine from the set of seven metabolites. Both programs also selected only one metabolite from the set of six metabolites with increased concentrations in the PICU sepsis cohort, with rDolphin identifying dimethylamine and BATMAN identifying lysine. The results for the Expert Profiler also showed that only three of the six metabolites in the increased concentration list were selected compared to all of the metabolites in the decreased concentration set. Since all of these results are based on how frequently metabolites were selected in resampled data sets in the BioMark program, this suggests that increased concentrations of some unselected metabolites might vary in this data set. We compared the ratios of the means for each metabolite to the results from the modelling results in Figure 2b but did not find a consistent explanation (see Supplementary Materials Table S1).

Precision results based on the number of correct identifications out of all selected metabolites by the BioMark program were all similar. The BioMark program settings were set to a high level of reproducibility, so this finding that about 30–60% of the biomarkers selected were the correct ones across all three methods suggests that some additional, individual metabolites might be important in this different approach. However, the identification of the list of true biomarkers was based on OPLS-DA that can take the correlation between metabolites into account, which may explain this finding. Lastly, the testing accuracy from the Multilayer Perceptrons with Hidden Multipliers (MLPHM) neural net found the manual profiling to be very accurate whereas the two automated methods were poor. This may be due to unclear features and relatively large amount of noise in these data sets obtained by the two automated methods, indicating that the quality of the metabolite concentrations was not as good. Overall, these findings suggest that neither rDolphin nor BATMAN could perform reasonably well compared to manual profiling.

Automated open-source programs have a number of important advantages for analysing NMR-based metabolomics data over manual profiling using a commercial program. First, these programs can provide greater consistency between experienced spectroscopists and laboratories, as the results should be highly reproducible using the same input data and parameters. Second, the time required to obtain metabolite concentrations is a matter of seconds up to a few minutes compared with 30–60 min for each ^1^H-NMR spectrum when performed manually. This gain in analysis time can be important when clinical decisions for treatment need to be made quickly, as in the case of sepsis [19]. Third, greater accuracy over time can be achieved, as manual curve fitting can be tedious and performance can drift due to the repetitiveness of the process. Less experienced users can use these programs with limited training or expertise. Lastly, open-source programs are free, so available study resources can be used for other expenditures rather than obtaining commercial licenses.

However, these open-source, automated programs also come with a number of disadvantages. They are not fully automated, as there still is some input required by the analyst that affects the identification and quantification of the spectra. In rDolphin, for example, an analyst could adjust the spectral fit based on visual assessment and error messages. This could improve performance but at the cost of extra time and the need for an expert profiler. Automated programs are also not as accurate as an expert who can match reference peaks found in the library to what is found in each sample and who can take into account overlapping peaks or superimposition of unknown signals. Based on our experience in the early phase of this project, a number of these open-source programs were not being maintained, would not install on our laptop for multiple reasons, including missing program components, or were no longer available to download. If workflows are built based on a program that is not maintained over time, then consistency and time-savings are not long-lived. The BATMAN program also required substantially more computational resources that might not be available to all users.

In conclusion, this pilot project aimed to answer the question: Can automated, publicly available programs that carry out identification and quantification of ^1^H-NMR metabolomics spectral data implemented by naïve users achieve acceptable results compared to those obtained by an expert? Our results for this pediatric sepsis data set of serum samples showed that neither the rDolphin nor BATMAN programs performed acceptably well. There might be several reasons for these discrepancies, including program capabilities as well as differences in the pre-processing of the spectra or in the adjustments or lack of adjustments made by human operators. We are investigating why each of the automated methods performed better with the decreased metabolite concentrations to better understand the differences in their approaches regarding quantification as implemented in this study. Automated approaches will not likely replace manual profiling by an expert, but they could be used to quickly determine whether known metabolites distinguish groups of patients. Newer, automated methods are being developed to overcome identification and quantification challenges, so the performance gap is likely to keep closing.

## 4. Materials and Methods

### 4.1. Study Data Set

Data utilized in this study were from a previous report that developed a biomarker panel based on metabolomics and inflammatory markers for the purposes of an early diagnosis and then triage of suspected pediatric sepsis cases [18]. In brief, children 2–17 years of age presenting with a diagnosis of sepsis at a pediatric intensive care unit (PICU cohort, i.e., required PICU care) or a pediatric emergency department (ED cohort, i.e., met sepsis definitions while in the ED but did not require PICU care) provided a blood sample for this study. Serum samples from 175 children were processed with a Bruker AVANCE-II 600 MHz spectrometer (Bruker BioSpin, Milton, ON, Canada) generating ^1^H-NMR spectra.

In the previous study, the spectra were manually analyzed by an expert (B.M.) with more than 10 years’ experience analysing NMR metolomics data. B.M. used Chenomx NMR Suite version 7.5 software (Chenomx, Edmonton, AB, Canada) which utilizes signal curves consisting of Lorentzian peaks based on a comprehensive database of metabolites used for manual profiling. Firstly, the ^1^H-NMR spectra were preprocessed, including phasing, removal of the water region, and baseline correction. Next, metabolite profiling was carried out with a concentration of 4,4-dimethyl-4-silapentane-1-sulfonic (DSS) acid used as a reference. Metabolite NMR chemical shift assignments were confirmed using 2D spectroscopy approaches—total correlation spectroscopy and ^1^H,^13^C heteronuclear single quantum coherence spectroscopy—and then compared with HMDB (version 2.5). A total of 58 metabolites were identified and quantified using this approach.

### 4.2. Preprocessing

In the current study, the ^1^H-NMR spectra preprocessing steps described above were carried out using Mnova v.14 [20], as the Chenomx preprocessed results were not available. Two samples were removed because one could not be pre-processed successfully and because for the other the sex of the child was not given. After uploading each raw Bruker file into Mnova, the water signal was removed followed by phase and baseline corrections and then peak alignment (see Supplementary Materials Pre-processing and Processing Steps). Training by the expert spectroscopist on the preprocessing steps was carried out for 10 spectra in the Mnova program, ensuring the naïve user obtained accurate data to input into the automated programs.

### 4.3. Metabolite Identification and Quantification

A total of 173 samples were submitted to rDolphin and BATMAN for targeted profiling. The expert spectroscopist ensured that the information required to run each program was correctly specified for the pilot runs and suggested adjustments if needed (e.g., ROI edges). Processing for each program was different. rDolphin input files included all 173 preprocessed spectra, and a .csv file with key information, such as ROI edges, metabolite names, and HMDB codes, chemical shifts and their tolerance, and J-coupling with the multiplicities (See Supplementary Materials Parameter input file example for rDolphin and Table S2). Several different ROI widths were evaluated to determine optimal ROIs in order to minimize fitting errors. The parameter .csv file provided options for normalization, alignment, and biofluid specification. Output from rDolphin included .csv files of the quantification, chemical shifts, fitting errors, intensities, ROI profiles used, signal area ratio, and half bandwidths. For the BATMAN package, key input files included an option file, a user file with the ppm position and multiplet information, a metabolite list, and processed spectra (see Supplementary Materials Parameter input file example for BATMAN and Table S3). A spectra plot of median concentrations can be useful to visually compare patient group values (Figure S1). BATMAN output files showed the posterior means of relative concentrations for the fitted metabolites and spectra, as well as their 95% credible intervals and information on the wavelet fit for unknown metabolites. The concentrations from each of the two approaches were scaled by sample-specific DSS concentrations and transformed by taking the natural logarithm with 0.0001 imputed for missing values.

### 4.4. Performance Metrics

The R package BioMark [21] was used to identify the significant biomarkers from metabolite concentrations obtained by rDolphin, BATMAN, and from the expert profiler. The BioMark package implements several classification methods, including principal component regression (PCR) and partial least squares-discriminant analysis (PLS-DA), as well as common selection methods, such as variable importance in projection (VIP) scores from PLS-DA models, Student’s *t*-tests, and the LASSO [22,23,24,25]. Two secondary selection methods are stability selection and higher criticism (HC) [26,27,28]. Stability selection identifies biomarkers in the top fraction of all available ones following many perturbations of the data, whereas HC compares the observed *p*-values with a uniform distribution under the null hypothesis to identify possible biomarkers. BioMark settings utilized in this study were unit variance scaling and log transformation for metabolite concentrations, Student’s *t*-tests for ranking, resampling using 70% samples and 50% variables per data set, and stability-based selection of 0.7 (a variable needed to be in the top 10 list at least 70 times out of 100 resampled data sets). These BioMark settings were selected based on evaluation of the concentrations obtained from manual fitting by a spectroscopy expert.

The true biomarkers were deemed to be the top six increased and top seven decreased metabolite concentrations based on the estimated regression coefficients from an orthogonal PLS-DA (OPLS-DA) model of the Chenomx metabolomics data for the 2–17-year-old children shown in Table 2 (unpublished results). These true biomarkers were selected based on the strength of their estimated regression coefficients and the fact that the lower bound of the confidence intervals was away from zero. In essence, they were the most robust metabolites in this data set that could distinguish between the two patient groups, but their individual clinical significance is unknown.

Performance metrics used to compare the two automated approaches with manual fitting included sensitivity (number of true biomarkers selected out of 13 true biomarkers), specificity (number of non-biomarkers selected/(58-13)), the area under the receiver operating characteristic curve (AUC—aggregate performance measure for classifying patients at various classification thresholds), and precision. Precision was defined as the number of true biomarkers selected out of the total number of metabolites identified by the BioMark program. A recently developed neural network was used to evaluate accuracy of predicting group assignment [29]. The MLPHM method uses a gate function that is applied to the hidden layers to reduce the number of hidden nodes and can incorporate noise to perturb the weights. The MLPHM method without noise weights is the version which was implemented here on the training subset (80% of the data set) and then on the testing data subset for each targeted approach. Group prediction, which was based on correctly classifying each sample to the PICU or ED sepsis group, on the average of 10 runs, provided the testing accuracy measure after the neural network had been trained. Run times for all methods were also measured.

## Figures and Tables

**Table 1:** Sensitivity, specificity, precision, and accuracy of each method (Expert Profiler, rDolphin and BATMAN) based on results from the BioMark Package and the Multilayer Perceptrons with Hidden Multipliers (MLPHM) algorithm.

Method	Sensitivity	Specificity	Precision	Testing Accuracy
Expert Profiler	76.9%	84.4%	62.5%	88.6%
	10/13	38/45	10/16	
Dolphin	30.8%	77.7%	30.8%	48.9%
	4/13	35/45	4/13	
BATMAN	30.8%	86.7%	44.4%	58.3%
	4/13	39/45	4/9	

**Table 2:** Top metabolites with increased/decreased relative concentrations in the PICU sepsis cohort samples compared to the pediatric emergency department (ED) sepsis cohort samples based on the ranked significant regression coefficients obtained from an orthogonal partial least squares-discriminant analysis OPLS-DA model.

Increased Concentration in PICU Cohort	Mean (SD^1^) PICU Sepsis	Mean (SD ^1^) ED Sepsis
Mannose	53.0	31.6
	(22.2)	(13.0)
Lysine	44.0	37.1
	(20.9)	(12.7)
Dimethylamine	1.58	0.77
	(1.76)	(0.46)
2-Hydroxyisovalerate	7.2	3.6
	(5.38)	(1.92)
Histidine	26.7	22.0
	(12.7)	(6.27)
Creatinine	49.3	28.1
	(47.5)	(16.2)
**Decreased concentration in PICU cohort**		
	
Acetate	25.5	43.5
	(14.3)	(22.2)
Taurine	28.6	47.9
	(19.9)	(25.7)
O-Acetylcholine	0.82	1.09
	(0.46)	(0.48)
Dimethyl sulfone	1.93	2.69
	(1.19)	(1.34)
Serine	38.8	49.5
	(16.4)	(15.2)
Citrate	39.2	46.4
	(37.6)	(17.3)
Alanine	92.9	119.3
	(70.3)	(47.9)

^1^ SD = standard deviation.

## Data Availability

The datasets analyzed during the current study are not publicly available due to limitations of consent for the original study but could be available from Joffe on reasonable request.

## Supplementary Materials

The following supporting information can be downloaded at: https://www.mdpi.com/article/10.3390/metabo12030227/s1, Ratio of Means and Table S1: Ratios of means of PICU sepsis patients to means of ED Sepsis patients; Pre-processing and processing steps; Materials Parameter input file example for rDolphin and Table S2: A portion of an example of an input RML file including ROI’s for rDolphin; and Figure S1: Median spectra from the rDolphin package comparing the PICU patients = 1 (blue) to the ED patients = 0 (orange) over the ppm range evaluated; Materials Parameter input file example for BATMAN and Table S3: A portion of an example of an input file multi_data_user for BATMAN.
