# Supplementary material for: Comparison of Two Automated Targeted Metabolomics Programs to Manual Profiling by an Experienced Spectroscopist for 1H-NMR Spectra
Source: Metabolites. 2022 Mar 4;12(3):227. doi: 10.3390/metabo12030227 (PMC8949809; doi:10.3390/metabo12030227)
Supplement: Supplementary file 1 [file metabolites-12-00227-s001.zip › metabolites-1534615-supplementary.pdf]

*metabolites*

MDPI

*Article*

# Comparison of Two Automated Targeted Metabolomics Programs to Manual Profiling by an Experienced Spectroscopist for $^{1}$H-NMR Spectra

**Xiangyu Wang [1], Beata Mickiewicz [2], Graham C. Thompson [3], Ari R. Joffe [4], Jaime Blackwood [5], Hans J. Vogel [6,*] and Karen A. Kopciuk [7,8]**

## Supplementary Materials

## Results

### Ratios of means

Since the means and standard deviations by each method before the same normalization had been applied were not available, the ratios of the means of the PICU Sepsis group to the means of the ED Sepsis groups were calculated for each metabolite. We compared these to Figure 2b results to see if they provided any insights; however, these results did not provide any consistent explanations. These are our findings from comparing the ratios to Figure 2b results:

***Mannose:*** Although these three methods have a similar ratio (>1), they all fail to find this metabolite.
***Lysine:*** rDolphin and Expert Profiler have essentially the same ratio, however, they both fail to find this metabolite. Surprisingly, BATMAN finds it.
***Dimethylamine***: BATMAN and Expert Profiler have a really similar ratio (>1), but both of them fail the find this metabolite. rDolphin does find this one, and its ratio is smaller than 1.
***2-Hydroxyisovalerate***: Only the Expert Profiler finds this metabolite, probably due to its lager ratio.
***Histidine:*** we cannot suggest a plausible reason for this one.
***Creatinine:*** Only the Expert Profiler finds this metabolite, probably due to its larger ratio.
***Acetate:*** All three methods can find this metabolite, no matter the ratio values.
***Taurine:*** rDolphin and BATMAN fail to find this metabolite with their higher ratio values (>1).
***O-Acetylcholine.:*** rDolphin and BATMAN fail to find this metabolite with their higher ratio values (>1).
***Dimethyl sulfone:*** we cannot suggest a plausible reason for this one.
***Serine:*** rDolphin and Expert Profiler find this metabolite, and have a very similar ratio values, while BATMAN doesn't (>1).
***Citrate:*** All three methods can find this metabolite, with all ratio values < .
***Alanine:*** Expert Profiler and BATMAN have very similar ratio values (both of them <1), and they find this metabolite. While, rDolphin fails to find it with its larger value.

**Table S1.** Ratios of means of PICU sepsis patients to means of ED Sepsis patients.

| **Increased Conc.** | **Methods** | **PICU Sepsis/ ED Sepsis** |
|---|---|---|
| Mannose | Expert Profiler | 1.6772 |
| | rDolphin | 1.0690 |
| | BATMAN | 2.0498 |
| Lysine | Expert Profiler | 1.1860 |
| | rDolphin | 1.1842 |
| | BATMAN | 3.3429 |
| Dimethylamine | Expert Profiler | 2.0519 |
| | rDolphin | 0.7231 |
| | BATMAN | 1.8245 |
| 2-Hydroxyisovalerate | Expert Profiler | 2.0000 |
| | rDolphin | 0.7857 |
| | BATMAN | 1.6933 |
| Histidine | Expert Profiler | 1.2136 |
| | rDolphin | 1.0956 |
| | BATMAN | 1.5237 |
| Creatinine | Expert Profiler | 1.7544 |
| | rDolphin | 0.8961 |
| | BATMAN | 1.6385 |
| Decreased Conc. | methods | PICU Sepsis/ ED Sepsis |
| Acetate | Expert Profiler | 0.5862 |
| | rDolphin | 0.4416 |
| | BATMAN | 1.1068 |
| Taurine | Expert Profiler | 0.5971 |
| | rDolphin | 1.2366 |
| | BATMAN | 1.2651 |
| O-Acetylcholine | Expert Profiler | 0.7523 |
| | rDolphin | 1.0100 |
| | BATMAN | 1.5716 |
| Dimethyl sulfone | Expert Profiler | 0.7175 |
| | rDolphin | 0.5036 |
| | BATMAN | 1.3521 |
| Serine | Expert Profiler | 0.7838 |
| | rDolphin | 0.7938 |
| | BATMAN | 1.4377 |
| Citrate | Expert Profiler | 0.8448 |
| | rDolphin | 0.7545 |
| | BATMAN | 0.6786 |
| Alanine | Expert Profiler | 0.7787 |
| | rDolphin | 1.1906 |
| | BATMAN | 0.8837 |

# Methods

2

**Pre-processing and processing steps**

These are the pre-processing steps and settings used in MNOVA:

1. Remove the water signal. (See MestReNova Manual 7.3.4, pp. 127-128). Choose the “signal suppression” to remove the water signal.
2. Phase Correction (See MestReNova Manual 7.4, pp. 147-156). Choose the auto phase correction on console.
3. Baseline Correction (See MestReNova Manual 7.5, pp. 157-164). Choose to correct baseline manually; choose polynomial order to be 4
4. Peak Alignment.
The position of DDS should be at 0 ppm. Choose this and align the peak of DDS to be 0 ppm. Zoom in to align precisely.
5. Binning (See MestReNova Manual 7.11, pp. 177-179)
Choose the range that you want to analyze, full spectra was our option. For the “width of integral region”, set it to be 0.004ppm, and “method” should be Sum.
6. Save the spectra

# Parameter input file example for rDolphin.

| **Parameter** | **Value** |
|---|---|
| nmr folder path | |
| 1D data index | |
| proc_no | |
| spectra dataset path (csv format) | C:\Users\input\spectra.csv |
| Metadata path (csv format) | C:\Users\input\MeTa.csv |
| ROI patterns file | C:\Users\input\RML.csv |
| Normalization (0=No;1=Eretic; 2=TSP; 3=Creatinine; 4=Spectra Sum; 5=PQN) | 5 |
| Alignment (0=No;1=Glucose; 2=TSP; 3=Formate) | 2 |
| Suppression | 5.1-4.5 |
| Spectrometer Frequency (MHz) | 600 |
| Bucket resolution | 0.004 |
| Biofluid | serum |
| 2D-Path | |
| Specific program parameters | |

# Parameter input file example for BATMAN.

%%%%%%%%%%%%%%%%%%%%%%%%%%%%%%%%%%%%%%%%%%%%%% Options file for BATMAN %%%%%%%%%%%%%%%%%%%%%%%%%%%%%%%%

%% There should be no empty line in the file, if line %%

%% is not used, please comment the start of the line with %. %%

%% Please do not comment or remove any of the parameter input lines. %%

%%%%%%%%%%%%%%%%%%%%%%%%%%%%%%%%%%%%%%%%%%%%%%%%%%%%%%%%%%%%%%%%%%%%%%%%%%%%%%%%%%%%%%%%%%%%%%%%%%%%%%%%%%%%%%%%%%%%%%%%%%%%%%%%%%%%%%%%%

%%
%% General parameters:
%% ppm ranges for analysis: Please put each ppm range in a pair of parentheses in the same line,
%% separate start and end ppm values with a comma, and
%% separate each set of ppm range with space, e.g. (-0.05,0.05) (1.44,1.52)
ppmRange - ppm ranges for analysis: (-0.15,0.15) (0.8,1.05) (1.1,1.6) (1.68,2.05) (2.05,2.3) (2.3,2.6) (2.6,2.85) (2.85,3.1) (3.05,3.15) (3.15,3.35) (3.35,3.5) (3.45,3.63) (3.63,3.8) (3.8,4.0) (4.0,4.2) (4.2,4.3) (4.3,4.83) (5,5.3) (5.3,5.5) (5.7,6.1) (6.4,6.8) (6.9,7.4) (7.4,7.8) (7.8,8.6) (9,9.8)
specNo - Ranges of spectra number to be included (e.g. 1,3-4 etc.): 1-15
paraProc - No of parallel processes (multicores) (only 1 core will be used for single spectrum): 15
negThresh - Truncation threshold for negative intensities: -0.5
scaleFac - Intensity scale factor: 800000
downSamp - Down sampling factor: 3
hiresFlag - Save metabolite fit at resolution of original spectrum? (Yes - 1 / No - 0): 1
randSeed - Random number seed: 100025
nItBurnin - Number of burn-in iterations: 2000
nItPostBurnin - Number of post-burn-in iterations: 500
multFile - Choose template of multiplets file from options below: 2
%% 1, The default template of multiplets in multi_data.csv file,
%% 2, The user input template of multiplets in multi_data_user.csv file,
%% 3, Both the default and user input template of multiplets files.
%%
thinning - Save MCMC state in every ? iterations: 50
cfeFlag - Same concentration for all spectra (fixed effect)? (Yes - 1 / No - 0): 0
nItRerun - Number of iterations for batmanrerun: 5000
startTemp - Start temperature: 1000
specFreq - Spectrometer frequency (MHz): 600
%%
%% Uncatalogued (wavelet) component:
%% Hyper parameters for the global precision priors, lambda is inversely proportional to error variance.
%% lambda ~ Gamma(a,b/2) on wavelet coefficients:
a - Gamma-distributed with shape a: 0.00001
b - Gamma-distributed with scale b: 0.000000001
%%
%% Catalogued metabolite component: gamma is the peak width (full width at half max) of metabolite m.
%% The model for gamma is ln(gamma)= mu + nu_m, where mu is the spectrum-wide average log-peak width
%% and nu_m is a random effect for each metabolite deviating from mu.
muMean - Mean of prior on global peak width (mu) in ln(Hz): 0
muVar - Variance of prior on global peak width (mu) in ln(Hz): 0.1
muVar_prop - Variance of proposal distribution for mu in ln(Hz): 0.002
%% The mean of each prior on nu_m is 0.
%% Set variance of prior on peak width offset (nu_m) to 0 to turn off the random effect on peak width:
4

nuMVar - Variance of prior on peak width offset (nu_m) in ln(Hz): 0.0025

nuMVarProp - Variance of proposal distribution for nu_m in ln(Hz): 0.1

%%

%% Wavelet truncation: tau is a vector of truncation limits, which sets a lower bound on the wavelets

tauMean - mean of the prior on tau: -0.05

tauPrec - inverse of variance of prior on tau: 2

%%

%% Global prior on peak shift (truncated Gaussian)

%% Note: individual priors for each multiplet can be changed in the multi_data(_user).csv file

rdelta - Truncation of the prior on peak shift (ppm): 0.002

csFlag - Specify chemical shift for each multiplet in each spectrum? (chemShiftperSpectra.csv file) (Yes - 1 / No - 0): 0

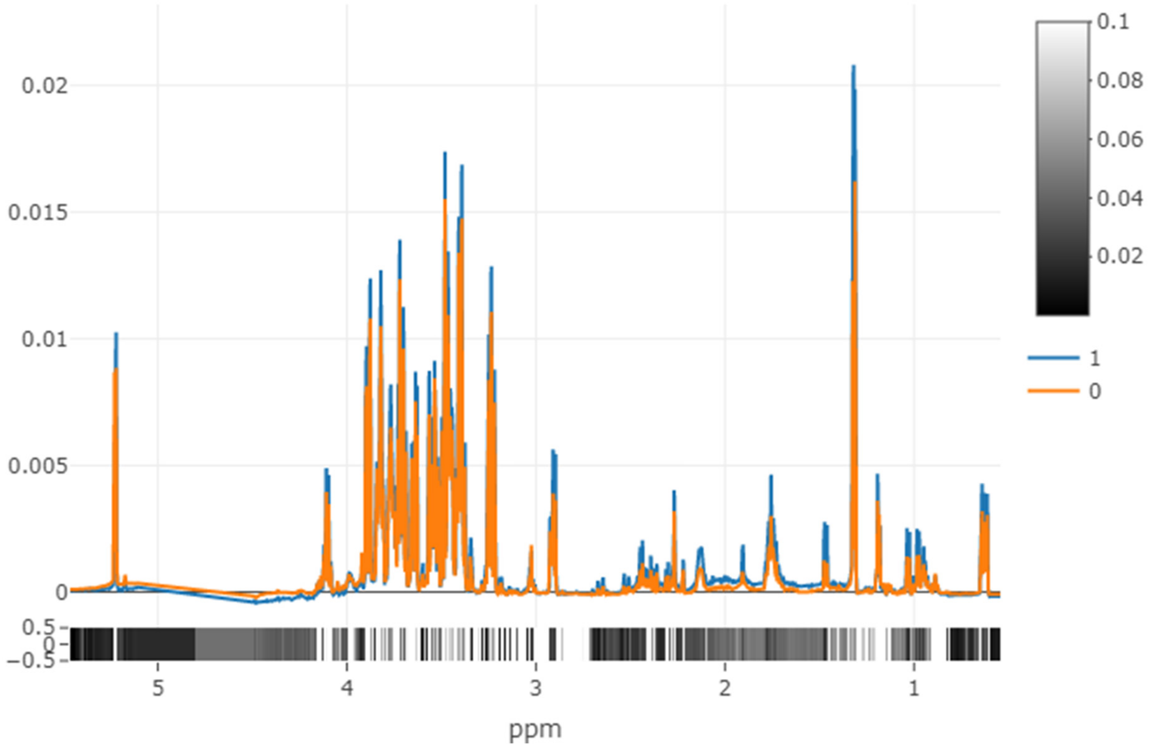


**Figure S1.** Median spectra from the rDolphin package comparing the PICU patients = 1 (blue) to the ED patients = 0 (orange) over the ppm range evaluated (note DSS is not included here).

**Table S2.** A portion of an example of an input RML file including ROI's for rDolphin.

| **ROI left edge** | ROI right edge | Quantifi-cation Mode | Metabolite | Quantifi-cation Signal | Chemical shift | Chemical shift tolerance | Half bandwidth | Multiplicity | J coupling | Roof effect | HMDB code |
|---|---|---|---|---|---|---|---|---|---|---|---|

| | | | | | | | | | | | |
|---|---|---|---|---|---|---|---|---|---|---|---|
| **0.04** | -0.04 | Clean Sum | DSS | 1 | 0 | 0.004 | 1.2 | 1 | 0 | 0 | |
| 0.84 | 0.8 | Baseline Fitting | 2-Hydroxyisovalerate | 1 | 0.821 | 0.005 | 1.2 | 2 | 6.87 | 0 | HMDB00407 |
| 0.9 | 0.85 | Baseline Fitting | Valproate | 1 | 0.863 | 0.008 | 1.8 | 3 | 8.655 | 0 | HMDB01877 |
| 0.9 | 0.85 | Baseline Fitting | 2-Hydroxybutyrate | 1 | 0.886 | 0.005 | 1.2 | 3 | 7.47 | 0 | HMDB00008 |
| 0.9 | 0.85 | Baseline Fitting | 3-Methyl-2-oxovalerate | 1 | 0.897 | 0.005 | 1.8 | 3 | 7.47 | 0 | HMDB00491 |
| 0.93 | 0.91 | Baseline Fitting | 2-Oxoisocaproate | 1 | 0.925 | 0.005 | 1.2 | 2 | 6.68 | 0 | HMDB00695 |
| 0.93 | 0.91 | Baseline Fitting | Isoleucine | 1 | 0.926 | 0.012 | 1.5 | 3 | 7.414 | 0 | HMDB00172 |
| 0.965 | 0.935 | Baseline Fitting | Leucine | 1 | 0.948 | 0.012 | 1.8 | 3 | 5.897 | 0 | HMDB00687 |
| 0.965 | 0.935 | Baseline Fitting | 2-Hydroxyisovalerate | 2 | 0.954 | 0.005 | 1.2 | 2 | 6.96 | 0 | HMDB00407 |
| 1.06 | 0.96 | Baseline Fitting | Valine | 1 | 0.976 | 0.008 | 1.2 | 2 | 7.01 | 0 | HMDB00883 |
| 1.06 | 0.96 | Baseline Fitting | 2-Aminobutyrate | 1 | 0.98 | 0.005 | 1.8 | 3 | 7.58 | 0 | HMDB00452 |
| 1.06 | 0.96 | Baseline Fitting | Isoleucine | 2 | 0.997 | 0.009 | 1.5 | 2 | 7.001 | 0 | HMDB00172 |

**Table S3.** A portion of an example of an input file multi_data_user for BATMAN:.

| Metabolite | pos_in_ppm | couple_code | J_constant | relative_intensity | overwrite_pos | overwrite_truncation | Include_multiplet |
|---|---|---|---|---|---|---|---|
| Alanine | 1.46 | 1 | 7.14 | 3 | 1.485 | n | 1 |
| Alanine | 3.76 | 3 | 7.2 | 1 | 3.787 | n | 1 |
| D-Glucose | 3.233 | 1,1 | 9.220,8.067 | 1 | 3.25 | 0.001 | 1 |
| D-Glucose | 3.524 | 1,1 | 9.823,3.732 | 1 | 3.5394 | 0.001 | 1 |
| D-Glucose | 3.889 | 1,1 | 12.293,2.140 | 1 | 3.901 | 0.001 | 1 |
| D-Glucose | 4.634 | 1 | 7.957 | 1 | n | n | 0 |
| D-Glucose | 5.223 | 1 | 3.677 | 1 | 5.2377 | 0.001 | 1 |
| Nicotinic acid | 7.51 | 1,1,1 | 7.91,4.99,0.72 | 1 | 7.529 | 0.001 | 1 |
| Nicotinic acid | 8.24 | 1,2 | 7.92,1.88 | 1 | 8.2584 | n | 1 |
| Nicotinic acid | 8.6 | 1,1 | 4.96,1.54 | 1 | 8.615 | 0.001 | 1 |
| Nicotinic acid | 8.93 | 1,1 | 2.15,0.72 | 1 | 8.944 | 0.001 | 1 |

| | | | | | | |
|---|---|---|---|---|---|---|
| Fumaric acid | 6.51 | 0 | | 2 | 6.522 | n | 1 |
| L-Glutamine | 3.766 | 2 | 6.18 | 1 | 3.781 | n | 1 |
| L-Glutamine | 2.148 | -2 | 2.09,2.206 | 2 | 2.149 | 0.001 | 1 |
| L-Glutamine | 2.451 | -2 | 2.38,2.522 | 2 | 2.4526 | 0.004 | 1 |
| Citric acid | 2.676 | -1 | 0,-15 | 0.7967,1.1799 | 2.678 | 0.003 | 1 |
| Citric acid | 2.558 | -1 | 0,-15 | 1.2242,0.7992 | 2.558 | 0.004 | 1 |
| DDS | 0 | 0 | 0 | 15 | n | n | 1 |
